# Diffuse alveolar hemorrhage in rheumatic diseases: Clinical features, diagnosis, and management

**DOI:** 10.1515/rir-2026-0008

**Published:** 2026-03-30

**Authors:** Nilanjana Dutta, Harshwardhan Patil, Vishnupriya Gopinath, Yashas Belliah Prakash, Sri Mounya Jampala, Naveenkumar Nallathambi, Mahabaleshwar Mamadapur

**Affiliations:** JSS Medical College, JSS Academy of Higher Education and Research, Mysuru, Karnataka, India; Department of Pharmacy Practice JSS College of Pharmacy, Mysuru, Karnataka, India; Vishnupriya Gopinath, J.S.S Medical College, J.S.S Academy of Higher Research, Mysuru, Karnataka, India; Adichunchanagiri Insitute of Medical Science, Adichunchanagiri University, BG Nagara, India; Sri Mounya Jampala, JSS Medical College, JSS Academy of Higher Education and Research, Mysuru, Karnataka, India; Naveenkumar Nallathambi, Institute of Internal Medicine Madras Medical College, Chennai, India; JSS University, JSS Academy of Higher Education and Research Mysore, Karnataka, India

**Keywords:** diffuse alveolar hemorrhage, pulmonary hemorrhage, systemic lupus erythematosus, ANCA-associated vasculitis, antiphospholipid syndrome, immunosuppression, rheumatic diseases

## Abstract

Diffuse alveolar hemorrhage (DAH) is a catastrophic pulmonary manifestation of systemic autoimmune and rheumatic diseases, particularly systemic lupus erythematosus (SLE), ANCA-associated vasculitis (AAV), and antiphospholipid syndrome (APS). It arises from immune-mediated injury to the pulmonary microcirculation, leading to intra-alveolar bleeding, respiratory failure, and high mortality. Despite advances in immunosuppression, DAH continues to pose major diagnostic and therapeutic challenges, with limited consensus on optimal management. We conducted an updated and comprehensive review of the epidemiology, pathogenesis, clinical spectrum, diagnostic strategies, therapeutic approaches, and outcomes of DAH in the context of rheumatic diseases through PubMed, Embase, and Scopus databases up to March 2025. DAH occurs in approximately 2%–5% of patients with SLE and up to 55% of patients with AAV, with reported mortality rates ranging from 20% to 80%. Hemoptysis is present in fewer than half of cases, highlighting the need for a high index of suspicion in patients presenting with anemia, dyspnea, and new bilateral infiltrates. Imaging typically reveals ground-glass opacities and consolidations on high-resolution CT, with a “crazy-paving” pattern observed during resolution. Bronchoscopy with bronchoalveolar lavage remains the diagnostic gold standard, confirming hemorrhage in sequential lavage aliquots. Histopathology demonstrates either pulmonary capillaritis or bland hemorrhage, with up to 73% of lupus-associated DAH cases showing the latter pattern without overt vasculitis. First-line therapy includes high-dose corticosteroids, frequently combined with cyclophosphamide or rituximab, while plasma exchange and intravenous immunoglobulin are considered in refractory disease. The PEXIVAS trial has challenged the universal role of plasma exchange, though it may retain a role in select patients with concomitant renal failure. Emerging therapies, such as complement inhibitors, B-cell targeted therapies, and mesenchymal stem cell transplantation, are under investigation. Supportive measures, including oxygen supplementation, noninvasive or invasive ventilation, and infection prophylaxis, are integral to management. Long-term survivors are at risk of recurrent hemorrhage and interstitial fibrosis. In conclusion, by consolidating current evidence and highlighting existing gaps, this review provides a clinically relevant framework to guide diagnosis, management, and future directions in the care of patients with DAH associated with rheumatic diseases.

## Introduction

Diffuse alveolar hemorrhage (DAH) is a severe and often life-threatening pulmonary complication arising from the pulmonary microcirculation, frequently encountered in systemic rheumatic diseases and associated with high morbidity and mortality.^[1]^ DAH is a distinct clinicopathologic syndrome characterized by bleeding from the pulmonary microcirculation, including alveolar capillaries, arterioles, and venules. Clinically, it manifests with hemoptysis, anemia, diffuse pulmonary infiltrates, and acute respiratory failure. Diagnosis is established by demonstrating the presence of red blood cells, fibrin, or hemosiderin-laden macrophages within the alveolar spaces on histopathologic examination.^[2]^ DAH is related to various immune and nonimmune conditions, with systemic lupus erythematosus (SLE), Granulomatosis with polyangiitis, and microscopic polyangiitis being the most common among immune disorders.^[3]^ Nonimmune causes include barotrauma, infection, inhalational injury, left ventricular dysfunction, renal failure, valvular diseases, *etc*. The occurrence of DAH in rheumatic diseases signifies heightened disease activity or a specific disease manifestation with potentially life-threatening consequences, including acute respiratory failure and death if not promptly recognized and treated.^[4]^ Although DAH has been described in association with several rheumatic diseases, the available literature is largely limited to case reports, small cohorts, and disease-specific reviews. A comprehensive synthesis that integrates epidemiology, clinicopathologic patterns, diagnostic strategies, therapeutic approaches, and outcomes across the spectrum of autoimmune conditions remains scarce. Our review addresses this gap by consolidating recent advances, including high-resolution imaging biomarkers, insights into distinct histopathologic subtypes such as capillaritis versus bland hemorrhage, and emerging immunomodulatory therapies beyond conventional corticosteroids and cyclophosphamide. We also highlight special clinical contexts such as pregnancy, pediatric patients, and infection-associated DAH that are often under-represented in prior reviews. By bringing together these diverse perspectives, this work provides a timely and clinically relevant framework for early recognition, individualized management, and future research directions in DAH associated with rheumatic diseases.

## Search Strategy

A comprehensive literature search was conducted to identify relevant studies on DAH in the context of systemic rheumatic diseases. Primary databases, including PubMed (MEDLINE, 2014–present), Scopus, and Embase, were systematically searched using keywords and controlled vocabulary (MeSH terms). The following search terms were used: “diffuse alveolar hemorrhage”, “DAH”, “pulmonary hemorrhage”, combined with disease-specific terms such as “systemic lupus erythematosus”, “SLE”, “ANCA-associated vasculitis”, “ANCA-associated vasculitis (AAV)”, “microscopic polyangiitis”, “granulomatosis with polyangiitis”, “Goodpasture syndrome”, “antiphospholipid syndrome”, “antiphospholipid syndrome (APS)”, “systemic sclerosis”, “rheumatoid arthritis”, “mixed connective tissue disease”, and “mixed connective tissue disease (MCTD)”. Boolean operators (AND/ OR) were employed to maximize sensitivity and specificity. MeSH terms included: “Pulmonary Hemorrhage”, “Vasculitis”, “Autoimmune Diseases”, “Rheumatic Diseases”, “Systemic Lupus Erythematosus”, and “Antineutrophil Cytoplasmic Antibody-Associated Vasculitis”.

Additional articles were identified through manual searches of Google Scholar, PubMed Central, and ScienceDirect, including the references of key review articles to enhance coverage. The selection process involved initial title screening and abstract and full-text review. Studies were included if peer-reviewed, published in English within the past 10 years, and focused on the clinical presentation, diagnosis, or management of DAH in rheumatic diseases. Exclusion criteria encompassed editorials, commentaries, non-English publications, and studies lacking relevant clinical data. Two independent reviewers performed the screening and data extraction, with disagreements resolved through discussion or consultation with a third reviewer. This structured methodology identified high-quality evidence to inform this narrative review.

## Pathophysiology of Diffuse Alveolar Hemorrhage

DAH is characterized by the extravasation of blood from the pulmonary microcirculation—primarily the alveolar capillaries, arterioles, and venules—into the alveolar spaces. This process does not originate from the larger airways or focal pulmonary lesions, distinguishing it from other causes of pulmonary bleeding.^[5,6]^ The classical clinical triad of DAH includes hemoptysis, new infiltrates on chest imaging, and anemia; however, hemoptysis is absent in a significant proportion of cases, underscoring the importance of integrating radiographic and laboratory findings to establish an accurate diagnosis. The central event in DAH pathogenesis is injury to the alveolar-capillary interface, particularly the basement membrane, resulting in increased vascular permeability and capillary leak. This damage may be driven by immune-mediated inflammation or direct vascular insult. In the context of autoimmune rheumatic diseases, dysregulated immune activation plays a critical role. A hyperinflammatory state—often described as a “cytokine storm” leads to the recruitment and activation of neutrophils, which release neutrophil extracellular traps (NETs), lytic enzymes, histones, and other proinflammatory mediators such as damage-associated molecular patterns (DAMPs).^[7,8]^ These factors perpetuate endothelial injury and amplify local tissue destruction.

Systematically, the underlying immunopathology varies among rheumatic diseases. In ANCA-associated vasculitis, small-vessel necrotizing vasculitis directly damages alveolar capillaries. In SLE, immune complex deposition and complement activation result in capillaritis and increased alveolar permeability. In contrast, APS –related DAH often stems from microthrombosis and secondary hemorrhage due to vascular occlusion, although vasculitis may also contribute. These mechanistic differences influence clinical presentation and therapeutic approaches, highlighting the importance of disease-specific evaluation. As the alveolar microvasculature becomes increasingly compromised, red blood cells leak into the alveolar spaces, impairing gas exchange and contributing to hypoxemia. The resulting intra-alveolar accumulation of blood can rapidly progress to respiratory failure if not promptly recognized and treated.^[9,10]^

**Figure 1:**
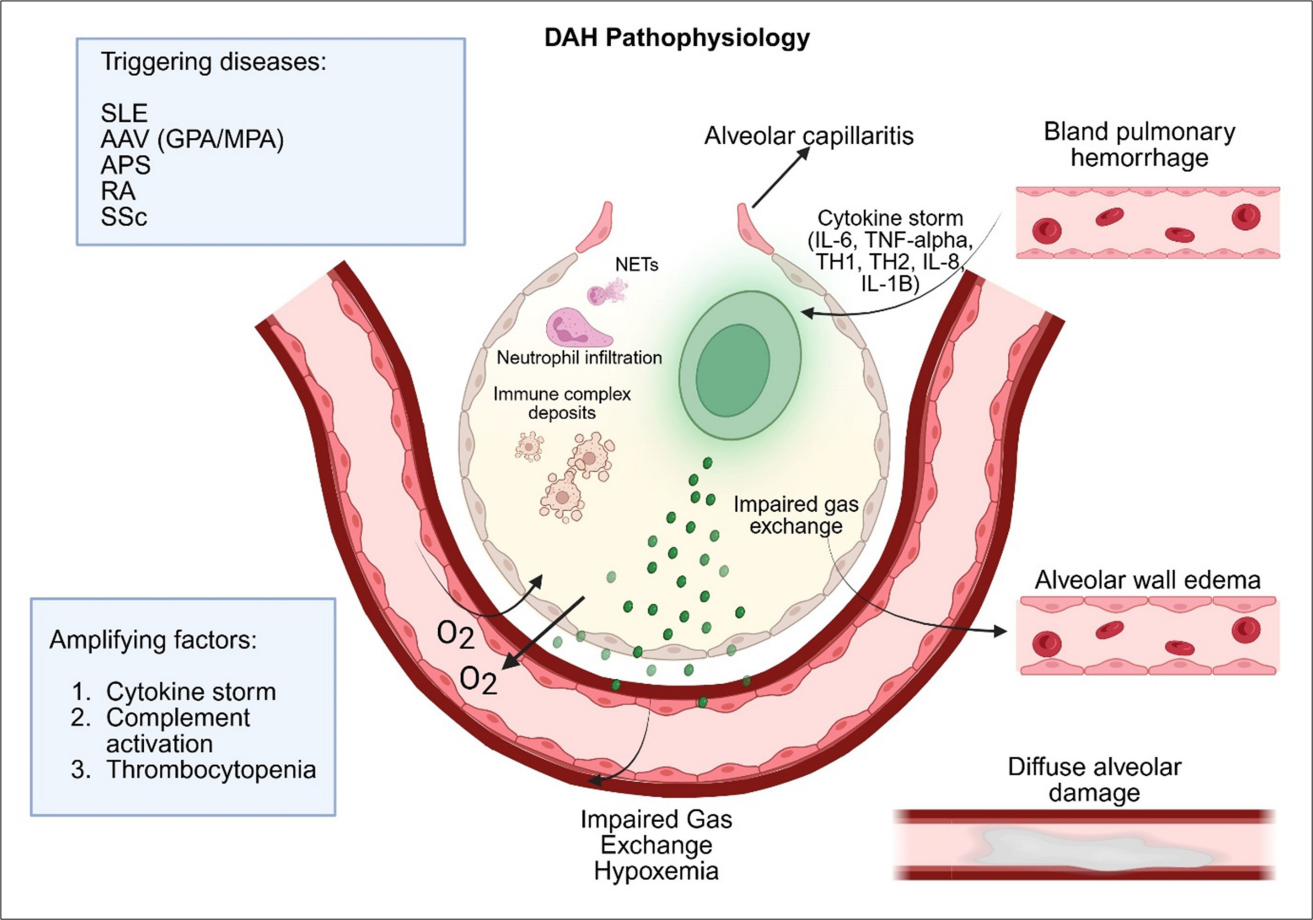
Pathophysiology of diffuse alveolar Hemorrhage. Patil, H. (2025). Pathophysiology of Diffuse alveolar Hemorrhage. Created in BioRender. https://BioRender.com/e7tujpf.

There are three main patterns of DAH, namely (Figure 1): (1) Pulmonary capillaritis is the most frequently encountered pattern and is characterized by the infiltration of neutrophils into the alveolar interstitium. This leads to damage or necrosis of the blood vessels and a subsequent loss of the structural integrity of alveolar epithelial and capillary endothelial cells.^[6]^ It is strongly linked to systemic vasculitides and various rheumatic diseases. (2) Bland pulmonary hemorrhage: characterized by bleeding into the alveolar spaces without significant inflammation or destruction of the underlying alveolar structures. This can be caused by anticoagulant therapy, idiopathic pulmonary, and mitral stenosis.^[5]^ (3) Diffuse alveolar damage: characterized by edema in the alveolar walls along with the formation of hyaline membranes. It is commonly associated with acute respiratory distress syndrome (ARDS), connective tissue diseases (including SLE), certain medications, and infections.^[5]^

## DAH In Rheumatic Diseases

### Systemic Lupus Erythematosus

The primary etiology of DAH in SLE is believed to be the deposition of immune complexes within the alveolar capillaries, leading to complement activation and inflammation. Both pulmonary capillaritis and bland hemorrhage have been observed.^[11]^ Risk factors include active lupus nephritis, high disease activity (high SLEDAI score), low complement levels, presence of antiphospholipid antibodies (especially lupus anticoagulant and IgG anticardiolipin), neuropsychiatric lupus, thrombocytopenia, and elevated C-reactive protein. A meta-analysis by González-Treviño consisting of nine studies encompassing 7746 SLE patients found that 2.1% developed DAH.^[11]^ In most patients, DAH succeeds a diagnosis of SLE, but it could the initial presentation of the disease as well. Many patients with SLE also have secondary anti-phospholipid antibody syndrome (APLA), which can also cause DAH.^[12,13]^ Serological workup in SLE–associated DAH often reveals high autoantibody titers and evidence of active nephritis.^[14]^ Histologically, two major patterns are described: pulmonary capillaritis, readily identified on light microscopy by inflammatory infiltration and vessel wall damage, and bland pulmonary hemorrhage (BH), which occurs without significant inflammatory changes. Interestingly, in one study, 73% of patients with lupus-related DAH demonstrated BH without capillaritis or cellular infiltration of the lung parenchyma. Notably, no clinical or radiographic differences were observed between patients with capillaritis and those with BH.^[13]^ Electron microscopy in cases of bland hemorrhage has revealed subtle endothelial abnormalities, supporting a microvascular basis for bleeding in the absence of overt inflammation.^[15,16]^

Another study reviewing admissions to a tertiary care centre reported an incidence of 0.52% among 1541 SLE patients.^[17]^ In children, DAH is a rare but serious complication of SLE. Acute pulmonary hemorrhage is one of the most severe forms of pulmonary involvement and occurs in less than 5% of children with SLE. Chang *et al*. found that dyspnea was the most common presentation, followed by haemoptysis ^11.^ Other presenting features include cough and hypoxemia^[18]^ Some studies suggest that thrombocytopenia in SLE may also suggest DAH, as reported by Kazzaz *et al*. in a cohort study conducted in the US.^[19]^

### Antiphospholipid Syndrome (APS)

DAH in APS results from microthrombi formation in the pulmonary microvasculature and/or pulmonary capillaritis induced by antiphospholipid antibodies (aPL). It is a rare but severe manifestation of APS, with reported prevalence ranging from 2% to 12%.^[20]^ In catastrophic APS, DAH has been reported in approximately 12% of patients.^[21]^ A history of thrombotic events or pregnancy morbidity and the presence of other autoimmune diseases like SLE are risk factors. Though pulmonary hypertension in APS is mainly an outcome of chronic pulmonary thromboembolism and vasculopathy, DAH without embolic disease can also lead to pulmonary hypertension.^[22,23]^ Pathogenesis includes aPL-induced endothelial activation, microvascular thrombosis, and complement activation. DAH can sometimes be the initial manifestation of APS, occurring in individuals without a prior diagnosis of the syndrome. Common symptoms include cough, dyspnea, fever, hemoptysis—hypoxemia, and anemia.^[24]^ Chest imaging shows diffuse alveolar infiltrates. Continual bleeding episodes can lead to mild to moderate interstitial fibrosis and restrictive lung disease. Bronchoscopy with bronchoalveolar lavage (BAL) confirms alveolar hemorrhage and testing for antiphospholipid antibodies is essential to confirm the diagnosis.^[25]^

### Anca-associated Vasculitides (AAV)

DAH in AAV is primarily due to pulmonary capillaritis, characterized by neutrophil infiltration and damage to alveolar capillaries, often mediated by antineutrophil cytoplasmic antibodies (ANCA), specifically PR3-ANCA in Granulomatosis with Polyangiitis (GPA) and MPO-ANCA in Microscopic Polyangiitis (MPA). Risk factors include genetic predisposition and environmental triggers like infections or toxins.^[5]^ Preexisting airway disease may also increase the risk. DAH is a more common complication in AAV compared to other rheumatic diseases. Mahajan *et al*. reported prevalence rates ranging from 7% to 45% in GPA^[24]^ and 10% to 55% in MPA.^[9,25]^ The PEXIVAS trial, the most extensive study in AAV, reported that 27.1% of participants had DAH.^[26]^ A recent single-center study of 133 AAV patients reported DAH in 31.5%, with a higher prevalence in MPA (37.05%) than GPA (15.5%).^[27]^ A retrospective cohort study by Kaneshita found that out of 123 patients with MPO-ANCA positive MPA, 30% had DAH.^[28]^ The clinical presentation of DAH in AAV is variable, ranging from subacute constitutional symptoms such as fever, malaise, anorexia, and arthralgia, which develop over days to weeks, to a rapid progression to severe respiratory failure.^[29]^ Risk factors for DAH were recognised as age, blood count, hematuria.^[30]^ Damage to the lung endothelial or epithelial cells and the entry of red blood cells into the alveolar cavity results from a breakdown in the integrity of the alveolar-capillary interface, resulting in decreased Hb.^[31]^ Most cases of AAV with DAH also have concurrent kidney involvement, with microscopic haematuria and proteinuria accounting for 97% and 79%, respectively.^[29]^ They also lead to decreased erythropoietin production leading to reduced Hb levels.^[32]^

Additionally, upper airway symptoms like sinusitis and nasal ulcerations are frequently encountered in GPA. Chest imaging shows diffuse bilateral infiltrates. Bronchoscopy with BAL confirms alveolar hemorrhage and ANCA testing (PR3 and MPO) is paramount for diagnosis.^[31]^ Common medications that have been implicated to cause AAV include propylthiouracil, hydralazine, sulfasalazine, anti-tumor necrosis factor alpha agents, D-penicillamine, and minocycline.^[33]^ Levamisole-adulterated cocaine has also been implicated in the develop-ment of ANCA.^[34]^ Approximately 50% of these patients demonstrate both MPO-ANCA and PR3-ANCA positivity.^[35]^

### Rheumatoid Arthritis (RA)

DAH in RA is rare and it occurs in association with vasculitis or capillaritis as in Wegener’s granulomatosis, Goodpasture syndrome, collagen vascular disease, microscopic polyangiitis and isolated pauci-immune pulmonary capillaritis, bland hemorrhages in patients with mitral valve involvement, overuse of anticoagulants, or due to drugs and toxins. Isolated case reports also reported DAH in RA patients being treated with Etarnacept and Amiodarone.^[36,37]^ While systemic vasculitis is usually associated with pulmonary capillaritis, it can also exist without involvement of other organ systems. Osman *et al*. reported such a case and also provided an overview of other such existing cases.^[38]^ Schwarz *et al* described 3 cases with a known diagnosis of RA who were reported to have DAH and capillaritis on lung biopsy, although without any associated systemic vasculitis or ANCA positivity.^[39]^ Seldomly, DAH can be the presenting feature of RA, and the prognosis depends on extent of pulmonary involvement, disease severity and treatment onset.^[39]^ Long-term care requires a multi-disciplinary approach to minimise relapse risk and optimise patient care. Complications include respiratory failure and even death, in severe cases. Corticosteroids and immunosuppressants remain the gold standard treatment with supplemental oxygen therapy, bronchodilators, and mechanical ventilation.^[40]^

### Systemic Sclerosis (SSc)

SSc is another rheumatic disease where DAH is an uncommon complication. Patients with SSc-associated DAH may present with nonspecific symptoms such as chest pain, cough, and dyspnea.^[41]^ It can be associated with capillaritis, systemic vasculitis, or bland pulmonary hemorrhage, or it can be an isolated occurrence with no related processes or conditions. DAH in SSc can progress to acute respiratory failure, necessitating prompt intervention. The etiology of Ssc can involve capillaritis, systemic vasculitis, or bland pulmonary hemorrhage. It can also be associated with scleroderma renal crisis.^[41,42]^ The first reported case of DAH in SSc was in 1977 by Kallenbach *et al*.^[43]^ Since then, very few reported cases are available in the literature. The nonspecific nature of the initial symptoms in SSc-related DAH underscores the importance of maintaining a high index of suspicion in SSc patients who develop new or worsening respiratory complaints, even if hemoptysis is absent.

### Inflammatory Myopathies

Polymyositis (PM) and dermatomyositis (DM) are rare but recognized causes of DAH, with only a few documented cases in the literature.^[24, 25, 26]^ These inflammatory myopathies typically present with symptoms such as muscle pain, proximal muscle weakness, elevated muscle enzymes (*e.g*., creatine kinase), and the presence of autoantibodies, such as anti-Jo-1 or other myositis-specific autoantibodies, in most cases. Amyopathic dermatomyositis (ADM), a form of DM lacking typical muscle involvement, has also been reported in association with DAH.^[44,45]^

Histopathological examination of lung biopsies from affected patients has revealed neutrophilic vasculitis and infiltration of the alveolar septa. Organizing pneumonia (OP) has been observed alongside DAH in several cases. However, it remains unclear whether OP is an initial manifestation of the underlying vasculitis or if it develops secondary to inflammatory processes, such as vasculitis or intra-alveolar hemorrhage. Pulmonary capillaritis is the most common manifestation in 80%–90% of the patients.^[46]^ It involves vasculitis, in which neutrophils infiltrate capillary walls causing necrosis and causing leakage of blood from the vessels into the alveolar space. Additionally, diffuse alveolar damage (DAD), characterized by hyaline membrane formation and fibrotic changes, has been identified in a cohort of patients with DM, indicating the presence of severe lung injury. Furthermore, thrombotic microangiopathy (TMA) has been reported in a patient with both DM and systemic sclerosis, further complicating the pathophysiology of DAH in these diseases. It is essential to highlight that while the association between inflammatory myopathies and DAH remains rare, these cases illustrate the complexity of managing rheumatic diseases with multisystem involvement, where the lungs may also be critically affected by immune-mediated damage. Table 1 summarises various case reports and review articles on DAH in rheumatic diseases.

**Table 1:** Summary of various cases and review articles on DAH in rheumatic diseases

Disease	Study	Findings
SLE	Amarnani (2021), Case series^[55]^	DAH reported as a rare but devastating complication.
	González-Treviño (2025), Meta-analysis^[56]^	Out of 7746 patients with SLE, 2.1% developed DAH.
	Chung (2009) - Retrospective study^[57]^	Out of 1541 SLE patients, 0.52% developed DAH.
	Kazzaz (2015), Case series^[20]^	Of the 22 patients with DAH, 59% were diagnosed with DAH within 5 years of SLE.
AAV	PEXIVAS trial (2024, Clinical Trials)^[26]^	Evaluated 704 patients with severe AAV; plasma exchange did not reduce the risk of death or end-stage kidney disease. A reduced-dose steroid regimen was non-inferior to standard dosing, with fewer serious infections.
	Berti (2021), Original article^[58]^	Found that DAH occurs in up to 25% of patients with AAV.
	Shiroshita, Meta-analysis^[60]^	DAH occurred in 10%-55% of MPA patients, 5%-30% of GPA patients, and 3%-8% of EGPA.
	Kida (2017), Retrospective cohort study^[61]^	Study of 123 patients with MPO-ANCA positive MPA Out of which, 30% had DAH.
	Yadavalli (2024), Review article^[27]^	Of 133 AAV patients, 31.5% had DAH—a higher incidence in MPA (37.05%) than GPA (15.5%).
APS	Stoots (2019), Cohort study^[62]^	In 483 primary APS patients, there is a 2% frequency of aPL-associated DAH.
Rheumatoid arthritis	Assad (2023), Case report^[37]^	Studied the relation between DAH in RA and amiodarone use. Found that DAH is a rare complication, possibly synergistically induced by amiodarone
	Saab (2019), Case report^[43]^	Reported a case of a patient with SSc and recurrent DAH.
	Osman (2017), Case report^[38]^	Reported a patient with RA and recurrent DAH.
Systemic Sclerosis	Da Silva (2022), Case report^[63]^	Reported a case of a patient with SSc and scleroderma renal crisis.
	Schwarz (1995), Case report^[39]^	Case of a 39-year-old male, Anti-Jo-1-antibody positive, case of PM, presented with acute respiratory failure.
Inflammatory myopathies	Samuel (2017), Case report^[46]^	Case of a 44-year-old male, Anti-PL-7 antibody, low complement, ANA with shortness of breath, intermittent hemoptysis, diagnosed with PM.
	Watanabe (2020), Case report^[42]^	82-year-old male, anti-MDA five antibody, diagnosed with DM.

This table summarizes representative studies and case reports highlighting the incidence, clinical characteristics, and outcomes of DAH across a range of systemic autoimmune and inflammatory rheumatic diseases. DAH, Diffuse alveolar hemorrhage SLE, Systemic lupus erythematosus; AAV, ANCA-associated vasculitis; MPA, Microscopic polyangiitis, GPA, Granulomatosis with polyangiitis; EGPA, Eosinophilic granulomatosis with polyangiitis; APS, Antiphospholipid syndrome, aPL, Antiphospholipid antibodies, RA, Rheumatoid arthritis SSc, Systemic sclerosis PM, Polymyositis, DM, Dermatomyositis, ANA, Antinuclear antibody MPO, Myeloperoxidase ESRD, End-stage renal disease.

### Special Considerations: Pregnancy, Pediatric, and Infection-Associated DAH

Pregnancy and childhood represent unique clinical contexts in which DAH in rheumatic diseases poses additional diagnostic and therapeutic challenges. In pregnant women, DAH may occur in association with SLE flares or APS, with maternal–fetal morbidity rates significantly higher than in non-pregnant cohorts. Studies stress the need for intensive care, higher rates of preterm delivery, and worsened maternal disease activity. Management therefore requires careful balancing of maternal stabilization and fetal safety, with high-dose corticosteroids being the immediate cornerstone therapy and Intravenous immunoglobulin (IVIG), plasmapheresis, and selective use of pregnancy-safer immunomodulators often used as steroid-sparing or rescue options when cytotoxic agents are contraindicated. These choices are further complicated in APS by the conflicting risks of thrombosis and bleeding and require individualized approaches to anticoagulation, immunomodulation, and plasma exchange.^[47,48]^ In pediatric patients, DAH is rare but often severe, with higher rates of respiratory failure and mortality compared to adults, underscoring the need for heightened vigilance and early bronchoscopy in unexplained anemia or hypoxemia.^[49,50]^ Recent pediatric cases document high rates of respiratory failure, repeated need for mechanical ventilation, and a significant mortality rate despite advances in supportive care, highlighting the need for early recognition, prompt bronchoscopy with BAL for diagnosis, and rapid escalation of immunosuppression or haemostatic therapies where indicated. Often children present with subtle signs (iron-deficiency anemia or unexplained hypoxemia) prior to haemoptysis, hence a low threshold for invasive diagnostic evaluation is recommended in the appropriate clinical settings.^[51]^ Furthermore, infections such as tuberculosis, influenza, and COVID-19 may precipitate or worsen DAH in rheumatic patients, either by amplifying systemic inflammation, worsening short term outcomes or by complicating immunosuppressive therapy. Recognition of these special scenarios is critical for optimizing outcomes and tailoring therapy in vulnerable populations.^[52–54]^

### Diagnosis of Diffuse Alveolar Hemorrhage

**Figure 2:**
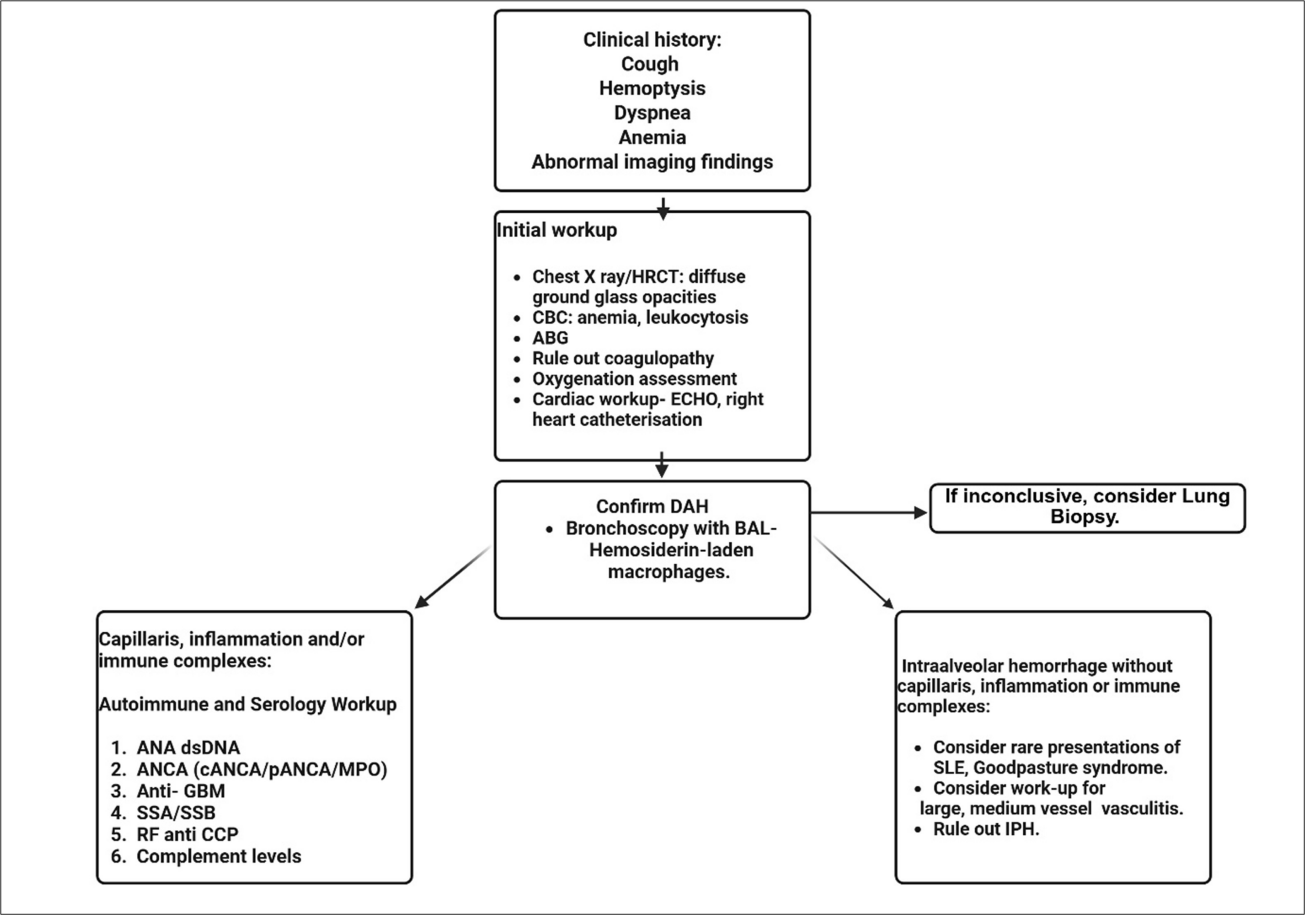
Approach to diagnosis of Diffuse alveolar hemorrhage in rheumatic diseases. Patil, H. (2025). Approach to diagnosis of DAH in rheumatic diseases. Created in BioRender. https://BioRender.com/kc1ktzb.

The diagnosis of DAH in patients with rheumatic diseases relies on a combination of clinical examination, findings from imaging studies, evidence of alveolar hemorrhage obtained through bronchoscopy with BAL, and supportive laboratory test results.^[6]^ Early and accurate diagnosis is essential for initiating timely treatment and improving patient outcomes. (Figure 2) Idiopathic pulmonary hemosiderosis (IPH) is a rare disease characterized by recurrent episodes of DAH without a known etiology, and this should be ruled out while diagnosing DAH in rheumatic diseases.^[44]^ DAH is a clinical syndrome with a diverse range of underlying etiologies, which can be broadly classified into immune-mediated, non-immune, and idiopathic causes. Pulmonary capillaritis is the most common histopathologic finding and is frequently associated with systemic autoimmune diseases such as vasculitides and connective tissue disorders. However, DAH can also result from diffuse alveolar damage, bland hemorrhage without inflammation, pulmonary vascular disorders, infections, drug exposures, and transplantation-related complications. A comprehensive understanding of the potential causes is essential for accurate diagnosis and timely management. The various etiologies of DAH are summarized in Table 2.^[1]^

**Table 2:** Etiological classification of DAH.

Category	Examples
Pulmonary Capillaritis	SLE, Goodpasture syndrome, Antiphospholipid syndrome, Granulomatosis with polyangiitis (Wegener), Microscopic polyangiitis, Mixed cryoglobulinemia, Behçet syndrome, Polymyositis, Scleroderma, Rheumatoid arthritis, Henoch-Schönlein purpura, Pauci-immune glomerulonephritis, MCTD.
Diffuse Alveolar Damage	SLE, Radiation therapy, Crack cocaine inhalation, Cytotoxic drugs, ARDS.
Bland Pulmonary Hemorrhage	Multiple myeloma, Subacute bacterial endocarditis, Goodpasture syndrome, Negative pressure pulmonary edema, Mitral stenosis, Coagulation disorders, Idiopathic pulmonary hemosiderosis, Drugs (see Table 2).
Other Autoimmune/Inflammatory	IgA nephropathy, Ulcerative colitis, Myasthenia gravis.
Transplant-related	Acute lung allograft rejection, Autologous bone marrow transplant.
Pulmonary Vascular Disorders	Pulmonary venous-occlusive disease, Pulmonary capillary hemangiomatosis.
Unclassified/Idiopathic	Idiopathic pulmonary fibrosis, Lymphangioleiomyomatosis, Unclassified

This table categorizes the diverse etiologies of DAH into clinically relevant groups based on underlying pathophysiologic mechanisms. Drug-induced causes are elaborated separately in Table 2. DAH, Diffuse alveolar hemorrhage; SLE, Systemic lupus erythematosus; MCTD, Mixed connective tissue disease; ARDS, Acute respiratory distress syndrome.

## Laboratory Tests

Lab tests are mainly used for identifying the underlying rheumatic disease causing DAH and are not specific to the condition. CBC can show anemia, which reflects blood loss into the lungs. Leukocytosis and increased inflammatory markers such as ESR and CRP may be present in cases of DAH associated with active inflammation, such as in vasculitis.^[7]^ Thrombocytopenia may also be present as a bleeding marker and should be evaluated. Urinalysis and measurement of serum creatinine levels are essential to assess for renal involvement, which is common in many rheumatic diseases causing DAH, such as SLE and AAV. Proteinuria, hematuria, and red blood cell casts in the urine can suggest systemic vasculitis or glomerulonephritis. ANA and rheumatoid factor may be positive in primary vasculitis, but high titers and disease-specific antibodies such as dsDNA, SS-A/SS-B, anti-ribonucleoprotein, and anti-JO-1 favor a CVD. Total IgE should be obtained when CSS is being considered. Complement (C3 and C4) should be obtained whenever SLE is in the differential.^[64]^ Anti-GBM is critical for identifying the underlying rheumatic disease and estimating complement C3 and C4 levels, which are low in certain diseases like SLE. Diffusing capacity for carbon monoxide (DLCO) may be a helpful test in cases of recurrent DAH; a restrictive pattern with a reduced DLCO may be observed, indicating lung fibrosis. Additionally, coagulation studies are usually performed to assess for any underlying bleeding disorders.^[5,6]^

## Imaging Studies

Chest radiography is usually the first test and is abnormal in most patients, showing new patchy or diffuse alveolar opacities; note that unilateral infiltrates are not rare (About 11% in a 112-patient series), and radiographic opacities may clear within 24–72 h once bleeding stops, which can mask early disease.^[65]^ However, these findings may be absent or nonspecific in milder cases. High-resolution CT (HRCT) is more sensitive and typically demonstrates ground-glass opacities (GGOs) with or without areas of consolidation; in a contemporary cohort of bronchoscopy-confirmed DAH, GGOs were present in 70.2% of patients.^[5,58]^ During the resolution phase of DAH, a “crazy-paving” pattern—ground-glass opacities with superimposed interlobular septal thickening—may be seen. In patients with recurrent DAH, HRCT can also show reticulation, suggesting evolving interstitial fibrosis. Additionally, HRCT is valuable for ruling out alternative causes of haemoptysis or diffuse lung infiltrates, such as infections or focal lung masses.^[6,58]^

## Bronchoscopy with Bronchoalveolar Lavage (BAL)

This is considered the gold standard for confirming the diagnosis of DAH and excluding other conditions, particularly pulmonary infections. In DAH, sequential lavage fluid samples will show an increasing number of RBCs, indicating active bleeding from the alveolar-capillary interface. The presence of hemosiderin-laden macrophages or siderophages in the BAL fluid, more than 20% or more of the cells retrieved, provides strong evidence of prior or chronic alveolar hemorrhage.^[66]^ If the diagnosis remains uncertain after bronchoscopy and BAL, or if a specific histopathologic diagnosis is needed to guide treatment, a lung biopsy may be performed.

To obtain a larger tissue sample for more specific histological evaluation, video-assisted thoracoscopic surgery may be necessary.^[7,41,67]^

## Treatment

### Pharmacological

Corticosteroids such as methylprednisolone are typically the first-line treatment for DAH. They are often administered intravenously in high doses (500–1000 mg/day for 3 consecutive days) followed by a gradual tapering of the oral dose. Corticosteroids act by suppressing pulmonary inflammation, stabilizing endothelial membranes, and reducing capillary permeability. Immunosuppressive agents are frequently used in conjunction with corticosteroids, particularly in DAH associated with capillaritis or severe underlying rheumatic disease. Cyclophosphamide, a potent cytotoxic and immunosuppressive agent, remains the standard for induction of remission in severe vasculitis and SLE-associated DAH. For systemic involvement, oral prednisone (1 mg/kg/day) in combination with oral cyclophosphamide (2 mg/kg/day) or intravenous pulse cyclophosphamide (10–15 mg/kg every 2–3 weeks) is recommended. Patients with renal impairment are treated with minimal effective doses, while elderly individuals receive reduced doses to mitigate toxicity. Alternatively, rituximab serves as an effective option, especially in ANCA-associated vasculitis and SLE, due to its favorable safety profile or in cases unresponsive or intolerant to cyclophosphamide. Other immunosuppressants, including mycophenolate mofetil, azathioprine, and methotrexate, may be used for maintenance therapy or disease-specific indications. IVIG can be considered in refractory cases of SLE- or APS-associated DAH.

**Figure 3:**
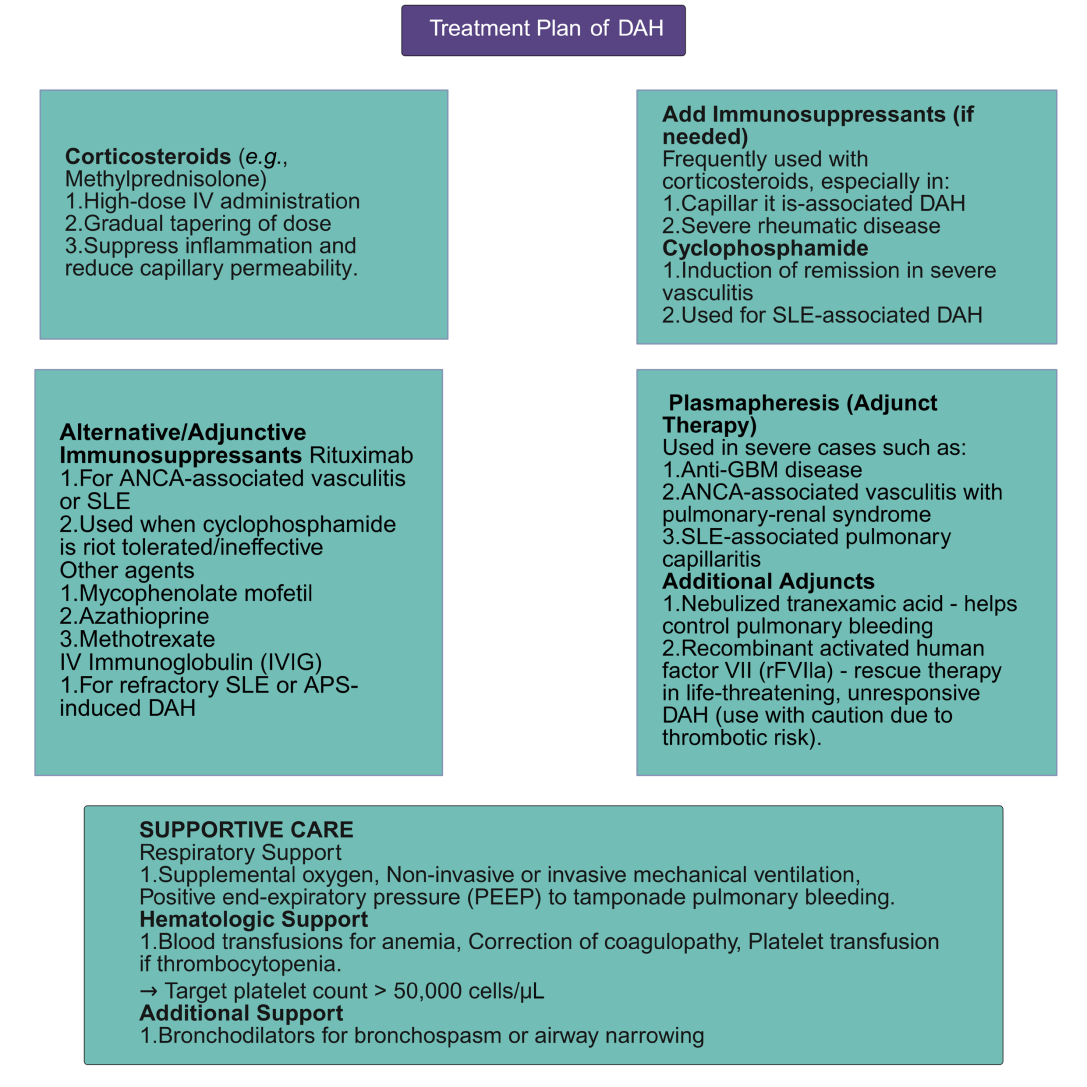
Treatment algorithm for diffuse alveolar hemorrhage (DAH).

In severe cases such as anti-GBM disease, ANCA-associated vasculitis with pulmonary–renal syndrome, and SLE-associated pulmonary capillaritis, plasmapheresis serves as an adjunctive therapy to remove pathogenic autoantibodies and inflammatory mediators. Nebulized tranexamic acid may be employed as an adjunct to control intrapulmonary bleeding, whereas recombinant activated human factor VII (rFVIIa) has been reported as a rescue therapy for life-threatening DAH unresponsive to standard interventions, although its utility remains uncertain due to potential thrombotic complications. In select cases, biologic therapies such as tocilizumab have been explored, particularly in connective tissue disease–related DAH, based on their capacity to modulate cytokine-driven inflammation. Supportive management—such as infection prophylaxis, oxygen supplementation, and ventilatory support—remains essential in all cases to optimize outcomes.^[13,58,59]^ Figure 3 provides a comprehensive plan for the treatment of DAH.

## Supportive Care

Supportive care includes providing supplemental oxygen, often through non-invasive or invasive mechanical ventilation, to maintain adequate oxygen levels in the blood. Positive end-expiratory pressure (PEEP) used during mechanical ventilation may help to tamponade the bleeding from the pulmonary capillaries. Blood transfusions are frequently necessary to treat the anemia resulting from the alveolar hemorrhage. While packed red blood cell transfusions can treat anemia in patients with DAH, they do not significantly improve tissue oxygen delivery or consumption. Bronchodilators may be used to alleviate any bronchospasm or airway narrowing that may be present. If hypotension occurs, intravenous (IV) fluids should be administered carefully to avoid the risk of pulmonary edema.^[68]^ Any underlying coagulopathy should be corrected, and thrombocytopenia should be managed with platelet transfusions if the platelet count is significantly low, with a target platelet count of greater than 50,000 cells per microliter.^[55,69]^

## Novel Strategies

Although controversial, the application of multipotent stem cells (MSCs) has been considered to treat various conditions, including graft-versus-host and autoimmune diseases. These cells augment various regulatory immune cells, including regulatory T and B cells, facilitating an anti-inflammatory environment.^[70]^ The therapy is given in addition to methylprednisolone and results in a good response. Oxygen saturation was improved, and complete resolution of lung infiltrates was seen after 2–3 weeks in SLE patients with DAH. Its application in other rheumatic diseases is still being explored.^[29]^ Given the limited efficacy and risks associated with conventional transfusions in the management of DAH, approaches such as thromboelastography (TEG) and rotational thromboelastometry (ROTEM) are gaining attention as promising tools. Evidence from research in cardiac surgery patients indicates that TEG/ ROTEM-guided transfusion protocols can reduce mortality and requirement of platelet, RBC, and fresh frozen plasma transfusions. TEG/ROTEM-guided transfusion could embody a valuable, targeted, and safer scheme for managing DAH in the future.^[71]^

## Prognosis, Long Term Outcomes, Recent Advances

The prognosis in patients with DAH due to rheumatic illnesses is quite severe, with in-hospital mortality rates described as ranging extensively from 20% to 100% based on the etiology, the severity of the hemorrhage, and with the presence of other organ involvement.^[3]^ In the context of SLE, mortality rates associated with DAH have been reported to be as high as 85.7% if treatment is delayed.^[71]^ For AAV, the first-year mortality rate in patients with DAH is estimated to be between 15% and 50%.^[20]^ Increased in-hospital mortality has been linked with the presence of shock, GFR below 60 mL/ min, cardiac failure, hypoxemia, aPL antibodies, and elevated levels of plasma LDH. Factors about increased long-term mortality include older age, the presence of cardiovascular comorbidities, and end-stage renal failure requiring hemo-dialysis.^[3]^ The need for mechanical ventilation to support breathing is also a significant indicator of a poorer prognosis.^[58]^ Recurrent episodes of alveolar hemorrhage can lead to long-term complications such as pulmonary hemosiderosis, pulmonary fibrosis, and rarely, emphysema, which can result in chronic pulmonary disability^[6]^ early recognition and prompt treatment help in improving survival rates and long-term outcomes in patients with DAH.

There are ongoing studies to further elucidate the association of DAH with various rheumatic conditions and to identify specific risk factors that may predispose patients to this severe complication. The PEXIVAS trial (Plasma Exchange and Glucocorticoids in Severe Antineutrophil Cytoplasmic Antibody–Associated Vasculitis)^[26]^ was a significant study that included patients with severe AAV, some of whom had DAH requiring mechanical ventilation. Subgroup analyses in patients with DAH indicated a trend towards possible benefit, even though the overall results did not demonstrate a significant reduction in mortality or end-stage renal disease with plasma exchange in the entire cohort.

Ongoing research also explores the use of targeted therapies, such as Rituximab, in treating DAH associated with rheumatic diseases.^[9]^ There is interest in investigating the role of novel hemostatic agents, such as intrapulmonary thrombin and recombinant activated human factor VIIa, in controlling the bleeding in severe cases of DAH. The impact of emerging infectious diseases, such as COVID-19, on the incidence and severity of DAH in patients with underlying rheumatic conditions is an area of growing concern and ongoing investigation.^[72]^ Future directions in research are likely to focus on developing more effective and targeted treatments for DAH in the context of different rheumatic diseases, as well as on improving our ability to predict which patients are at the highest risk of developing this serious complication.

## Future Directions

Future research should focus on unravelling the precise molecular and immunologic mechanisms underlying DAH in specific rheumatic diseases, identifying early biomarkers for risk stratification, and evaluating novel immunomodulatory and targeted biologic therapies through well-designed clinical trials. Collaborative registries and multicenter studies are also needed to establish evidence-based guidelines and improve long-term outcomes in this vulnerable patient population.

## Conclusion

This review underscores the complex definition, multifactorial pathophysiology, and broad spectrum of rheumatic diseases associated with DAH. It highlights disease-specific mechanisms, clinical heterogeneity, diagnostic challenges, therapeutic strategies, and patient outcomes. Timely diagnosis—facilitated by a high index of clinical suspicion, imaging studies, bronchoscopy with bronchoalveolar lavage, and laboratory evaluation—is essential for early intervention. Despite advances in therapy, DAH remains a life-threatening complication in systemic autoimmune diseases, with a high mortality rate.

In our study, DAH most commonly occurred early in the disease course and rarely presented with hemoptysis. The overall mortality approached 50%, even with aggressive immunosuppressive therapy. Our findings suggest that favourable outcomes may depend on the prompt initiation of plasmapheresis in combination with high-dose intravenous methylprednisolone and cyclophosphamide. There is a pressing need to refine therapeutic protocols, enhance predictive tools, and pursue personalized treatment strategies to improve prognosis in these critically ill patients.
